# Phytogenic Synthesis of Nickel Oxide Nanoparticles (NiO) Using Fresh Leaves Extract of *Rhamnus triquetra* (Wall.) and Investigation of Its Multiple In Vitro Biological Potentials

**DOI:** 10.3390/biomedicines8050117

**Published:** 2020-05-12

**Authors:** Javed Iqbal, Banzeer Ahsan Abbasi, Riaz Ahmad, Mahboobeh Mahmoodi, Akhtar Munir, Syeda Anber Zahra, Amir Shahbaz, Muzzafar Shaukat, Sobia Kanwal, Siraj Uddin, Tariq Mahmood, Raffaele Capasso

**Affiliations:** 1 Department of Plant Sciences, Quaid-i-Azam University, Islamabad 45320, Pakistan; benazirahsanabbasi786@gmail.com (B.A.A.); s.a.zahra786@gmail.com (S.A.Z.); amirqaisrani@gmail.com (A.S.); m.muzaffar.shoukat@gmail.com (M.S.); usiraj85@gmail.com (S.U.); tmahmood.qau@gmail.com (T.M.); 2 College of Life Sciences, Shaanxi Normal University, Xi’an 710119, China; riaz17qau@gmail.com; 3 Department of Biomedical Engineering, Yazd Branch, Islamic Azad University, Yazd 8915813135, Iran; m.mahmoodi@iauyazd.ac.ir; 4 Department of Chemistry and Chemical Engineering, SBA School of Science and Engineering, Lahore University of Management Sciences (LUMS), DHA, Lahore 54792, Pakistan; 16130031@lums.edu.pk; 5 Department of Zoology, University of Gujrat, Sub-Campus Rawalpindi 46000, Pakistan; sobiakanwal16@gmail.com; 6 Plant Breeding Institute, Faculty of Agriculture & Environment, University of Sydney, Cobbitty, NSW 2570, Australia; 7 Department of Agricultural Sciences, University of Naples Federico II, 80055 Portici, Italy

**Keywords:** *Rhamnus triquetra*, NiONPs, cytotoxicity, biocompatibility, antimicrobial, enzyme inhibition

## Abstract

Chemically nickel oxide nanoparticles (NiONPs) involve the synthesis of toxic products, which restrict their biological applications. Hence, we developed a simple, eco-friendly, and cost-efficient green chemistry method for the fabrication of NiONPs using fresh leaf broth of *Rhamnus triquetra* (RT). The RT leaves broth was used as a strong reducing, capping, and stabilizing agent in the formation of RT-NiONPs. The color change in solution from brown to greenish black suggests the fabrication of RT-NiONPs which was further confirmed by absorption band at 333 nm. The synthesis and different physicochemical properties of RT-NiONPs were investigated using different analytical techniques such as UV-Vis (ultraviolet−visible spectroscopy), XRD (X-ray powder diffraction), FT-IR (Fourier-transform infrared spectroscopy), SEM (scanning electron microscopy), TEM (transmission electron microscopy), EDS (energy-dispersive X-ray spectroscopy), DLS (dynamic light scattering) and Raman. Further, RT-NiONPs were subjected to different in vitro biological activities and revealed distinctive biosafe and biocompatibility potentials using erythrocytes and macrophages. RT-NiONPs exhibited potential anticancer activity against liver cancer cell lines HUH7 (IC_50_: 11.3 µg/mL) and HepG2 (IC_50_: 20.73 µg/mL). Cytotoxicity potential was confirmed using Leishmanial parasites promastigotes (IC_50_: 27.32 µg/mL) and amastigotes (IC_50_: 37.4 µg/mL). RT-NiONPs are capable of rendering significant antimicrobial efficacy using various bacterial and fungal strains. NiONPs determined potent radical scavenging and moderate enzyme inhibition potencies. Overall, this study suggested that RT-NiONPs can be an attractive and eco-friendly candidate. In conclusion, current study showed potential in vitro biological activities and further necessitate different in vivo studies in various animal models to develop leads for new drugs to treat several chronic diseases.

## 1. Introduction

Nanotechnology deals with different approaches to synthesize materials ranging from 1 to 100 nm, at least in one dimension, and have unique properties, such as small size, surface charge, porosity, high surface energy, and high surface area/volume (S/V) ratio, which enhance their catalytic properties and interaction with other molecules. The main reason why metal nanoparticles (MNPs) have gained the specific attention of researchers is due to their unique properties, namely particle size, shape, crystal structure, surface effect, magnetic, catalytic, optical, as well as chemical and mechanical characteristics from their bulk counterpart [1,2]. Until now, different multifunctional metals and metal-oxide nanoparticles have been synthesized [3,4]. Among the different nanoparticles(NPs), NiONPs have gained the specific attention of biologists and chemists due to their numerous applications in battery electrodes, magnetic materials, heterogeneous catalysts, gas sensors, electrochromic films, and solid-oxide fuel cells and help in the adsorption of inorganic pollutants and dyes [5,6]. Further, NiONPs have shown significant antibacterial, antifungal, antioxidants, anti-inflammatory, anticancer, and enzyme inhibition potentials [6,7]. NiONPs have shown toxicity towards different microbial agents and microalgae by producing ROS, inducing oxidative stress and releasing (Ni2+) inside the cell [8].

Currently, NiONPs are fabricated via different physical and chemical approaches. However, these synthesis routes face several challenges as they utilize costly metal salts, organic solvents, toxic reducing agents (sodium borohydrides, hydrazine hydrate, sodium citrate and Gallic acid), stabilizing and capping agents (thiols, amines, sodium citrate), and demand expensive equipment. These approaches are not only expensive at the industrial scale, but also cause some undesired effects on human life and the surrounding environment, and may result in cytotoxicity, carcinogenicity, and genotoxicity, thus restricting their utilization in biomedical purposes [9]. Hence, these problems must be solved, and actions are required to develop an alternative solution for the fabrication of NPs.

Therefore, scientists have developed green chemistry methods which are more sustainable, cleaner and eco-friendly. Presently, new developments have been made in the synthesis of nanomaterials using different biological sources (microbes, algae, fungi, various lower and higher plants). This method is relatively simple, ecofriendly, energy-efficient, nontoxic, eliminates the need for high energy, temperature, and pressure and needs no reducing, stabilizing, and capping agents from outside. The major disadvantages associated with microbial synthesis is the maintenance of an aseptic environment, culturing in media, high isolation cost, high incubation time, difficulty in handling, pathogenicity in nature, and the requirement of comprehensive biological knowledge [10].

Phytofabrication has flourished for the formation of several nanomaterials and has attracted the attention of the nano task force due to its sample, environmentally benign, and cost-effective nature [11,12,13,14]. In green synthesis, phytoconstituents (alkaloids, terpenoids, polyphenols, glycosides, flavanoids, proteins, vitamins, polysaccharides) function as a capping and reducing agent like different chemical substitutes used in the chemical synthesis of nanoparticles [15,16]. There are multiple factors that influences green synthesis of nanoparticles, such as nature of plant extracts, concentration of extracts, metal salt, pH, and synthesis protocol used. Thus, for the green synthesis of MNPs, 12 basic principles of green chemistry are now becoming a reference guideline for researchers, chemical technologists, and chemists worldwide to develop less dangerous chemical products and byproducts [17,18,19]. Therefore, green nanotechnology is an alternate route for the formation of safe and stable materials using different medicinal plants and thus has experienced for rapid rise [20,21,22,23].

In the present study, fresh leaves extract of *R. triquetra* were used to synthesize NiONPs. The plant is found in abundance in Pakistan (Kashmir, Margalla Hills), Nepal, and India during the summer season between July and August. The bark, leaves, and fruits of *R. triquetra* are used to treat hemorrhagic septicemia in livestock, intestinal worms, and malarial fevers, possessing significant antimicrobial, deobstruent, anti-inflammatory, astringent, and antioxidant properties. This plant contains several ecofriendly phytoconstituents such as emodin, Kaempferol-7-O-CH_3_ ether, Kaempferol-4-O-CH_3_ ether, gluside, quercetins, and physcion [24,25] which help in the phytofabrication of NiONPs. As per the available literature and knowledge, this is perhaps the first study reported on the green synthesis of NiONPs employing *R. triquetra* leaves broth. NiONPs were characterized using different characterization techniques. Further, considering biological and therapeutic potential of *R. triquetra*-NiONPs, different biological activities; anticancer, antimicrobial, antileishmanial, antioxidant, and enzyme inhibitory assays were performed.

## 2. Material and Methods

### 2.1. Preparation of R. Triquetra Leaf Extract

The preparation of *R*. *triquetra* leaves extract was achieved using previously optimized protocol [26]. Precisely, *R. triquetra* leaves were collected from Pir Suhawa Margalla hills Pakistan (33.7870° N, 73.1084° E). The sample was identified by senior taxonomist Dr. Syed Afzal Shah, Department of Plant Sciences, QAU Islamabad, Pakistan. The leaves were thoroughly washed with running tap water followed by washing with distill water. The leaves were shade dried and crushed into fine powder. Then, twenty gram leaves powder was added into 100 mL distill water and heated at 80 °C for 1 h. The resultant extract was filtered three time using Whattman filter paper No.1 (cone shaped), centrifuged at 5000 rpm for 20 min to remove all unwanted aggregates. Finally, the plant extract was stored at 4 °C till further use.

### 2.2. Green Fabrication of NiONPs

**Figure 1:**
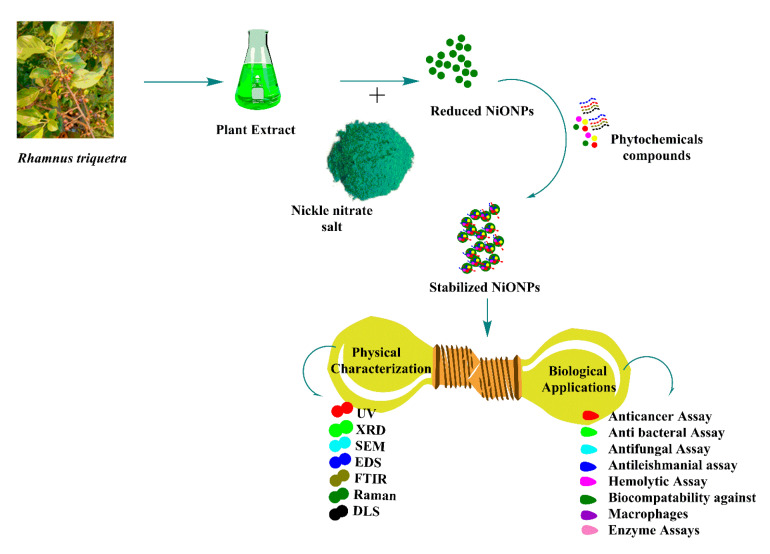
General overview of the study plan.

Synthesis of NiONPs was performed by reducing nickel nitrate using *R*. *triquetra* leaf extract. To achieve this purpose, 100 mL filtered RT leaves extract was steadily mixed with 1 gm NiNO_3_ salt followed by continuous heating (70 °C) and stirring at 500 rpm for 2 h to achieve homogeneous solution. Further, obtained solution was centrifuged at 4000 rpm/20 min. Supernatant was discarded and pellet containing NiONPs was carefully washed 3 times with distilled water to remove uncoordinated materials. The obtained powder assumed as NiONPs was incubated at ~100 °C until the water evaporated completely, followed by annealing. Further, NiONPs were stored in cool, dry, and dark place. Finally, NiONPs were thoroughly characterized. Figure 1 shows a schematic representation of the synthesis, characterization, and biological application of NiONPs.

### 2.3. Physical Characterizations of NiONPs

The physicochemical properties of RT-NiONPs were investigated using different analytical techniques. The optical properties and bioreduction of nickel ions to NiONPs was confirmed by measuring the absorption spectra of reaction solution using a UV-spectrophotometer, and the solution was scanned between 200 and 600 nm. DLS analyses has provided further insight into the average hydro-dynamic particles diameter (d. nm), ζ-potential and PDI of NiONPs using Malvern Zetasizer Nano (Malvern instrument). RT-NiONPs were analyzed by Fourier transform infrared (FT-IR) spectroscopy to detect different bioactive functional groups responsible for the synthesis and stabilizing NiONPs using various modes of vibrations. FT-IR measurement of the sample was scanned in the wavenumber region 500 cm^−1^ to 4000 cm^−1^. The structural analysis and crystalline nature of biogenic NiONPs was carried out using XRD analysis (PANalytical XRD (Netherland). The nano-crystallite size was calculated from the width of the XRD peaks using Debye-Scherrer’s equation. The vibrational characteristics of RT-NiONPs were studied using Raman spectroscopy. The elements of NiONPs were detected by EDX (energy dispersive X-ray). The morphological features (surface topology) of *R*. *triquetra*-NiONPs was studied using SEM (EM (NOVA FEISEM-450 applied with EDX detectors). In addition, the morphological structure and actual particle size was studied under TEM (transmission electron microscopy).

### 2.4. Antileishmanial Potentials (ALP)

The in vitro antileishmanial potential of NiONPs was investigated using MTT cytotoxicity assay [7]. To confirm the antileishmanicidal potential, *Leishmania tropica* “KWH23 strain” (promastigote and amastigotes parasites) was cultured in MI-99 media containing 10% FBS. The 200 µL reaction mixture is comprised of 100 µL of standardized culture, fresh media (50 µL) and colloidal nanoparticles (50 µL) suspension. Amphoterecin B served as positive while DMSO function as negative control. The leishmanial parasites *L*. *tropica* were kept in 96-well plate and were treated with different concentration of NiONPs (1100–8.595 μg/mL) to determine their antileishmanial potency. The test sample (NiONPs) was incubated in 5% CO2 incubator at 24 °C/72 h. After treatment and incubation with NiONPs, the reaction mixture was scanned at 540nm using micro-plate analyzer and readings were taken. Both parasites were counted and IC_50_ values were calculated to determine intensity/degree of antileishmanicidal potential using formula below:

(1) $$\%\mathrm{inhibition}=\frac{1-\mathrm{sample}\;\mathrm{absorbance}}{\mathrm{absorbance}\;\mathrm{of}\;\mathrm{control}}\times 100$$

### 2.5. Anticancer Activity

The in vitro anticancer potential of *R*. *triquetra* mediated NiONPs was investigated using HepG2 and HuH7 cancer cell lines using an MTT assay [27]. The cancer cells were cultured in flasks containing DMEM media supplemented with 10% FBS, Pen-Strep and kept in 5% CO2 incubator for 24 h/37 °C. The confluent HepG2 and HuH7 cancer cells (4000 cells/well) were carefully seeded in 96-well plate. Further, cells were treated with varying doses of RT-NiONPs (1100–8.595 μg/mL) for 48 h. DMEM media was removed and MTT solution (100 μL) was added in each well followed by further incubation (3 h in 5% CO2 incubator/37 °C). The DMEM media containing other components (FBS, Pen-Strep) was removed and DMSO (100 μL) was loaded in each well followed by incubation for ~20–30 min. The conversion of MTT solution to formazan by living cells was measured using micro plate analyzer 570 nm wavelength. The untreated cancer cells were considered as control and % inhibition of HepG2 and HuH7 cell lines exposed to different concentration of NiONPs was calculated:

(2) $$\%\mathrm{inhibition}=\frac{1-\mathrm{OD}\;\mathrm{of}\;\mathrm{sample}}{\mathrm{OD}\;\mathrm{of}\;\mathrm{control}}\times 100$$

### 2.6. Biocompatibility with Human Erythrocytes (RBCs) and Macrophages

To determine the non-toxic nature of NiONPs, hemolysis assay was done using erythrocytes cells as discussed in the previously published article [28]. To achieve this purpose, 1 mL freshly isolated human red cells was placed in an Ethylenediaminetetraacetic Acid (EDTA) tube to avoid blood coagulation. Further, erythrocytes were centrifuged at 12,000 rpm/10 min. After centrifugation, supernatant was discarded, and pellet was rinsed three times with PBS. Erythrocytes suspension was made adding 200 µL erythrocytes into 9.8 mL PBS. Further, erythrocytes suspension (100 µL) was treated with various concentration of NiONPs and reaction mixture was incubated at 36 °C for 1 h followed by centrifugation (12,000 rpm/15 min). Supernatant was transferred into 96-well-plate and release of hemoglobin was studied at wavelength (540 nm) using micro-plate analyzer. Triton X-100 was used as positive and DMSO as negative control respectively. The data obtained was calculated as % hemolysis caused by different doses of NiONPs using formula below:

(3) $$\%\;\mathrm{hemolysis}=\frac{\mathrm{Sample}\;\mathrm{abs}-\mathrm{Negative}\;\mathrm{control}\;\mathrm{abs}}{\mathrm{Positive}\;\mathrm{control}\;\mathrm{abs}-\mathrm{Negative}\;\mathrm{control}\;\mathrm{abs}}\;\times \;100$$

The biosafe nature of RT-NiONPs was further confirmed using human macrophages (HM) following previously used protocol [14]. To confirm the biosafe nature, HM cells were culture in flasks containing RPMI media provided with 10% FBS, Hepes, Pen-Strep (antibiotic). For the proper growth and attachment of cells, flasks containing macrophages were transferred in 5% CO2 incubator for 24 h. After culturing, macrophages (4000 cells/well) were loaded into 96-well-plate. After incubation, macrophages were treated with varying doses of NiONPs (1100–8.595 μg/mL). Finally, the % inhibition of HM cells treated with different doses of RT-NiONPs was calculated using the formula below:

(4) $$\%\;\mathrm{inhibition}=\frac{1\;-\;\mathrm{Absorbance}\;\mathrm{of}\;\mathrm{sample}}{\mathrm{Absorbance}\;\mathrm{of}\;\mathrm{control}}\;\times \;100$$

### 2.7. Antioxidant Activities

Spectrophotometric procedures were used to confirm the antiradical potentials of RT-NiONPs using Total antioxidant capacity (TAC), CUPARAC, DPPH, and total reducing power (TRP). Total antioxidant capacity (TAC) was determined through the phosphomollybdenum method [29]. The incubation of RT-NiONPs with Molybdenum (VI) demonstrated the presence of antioxidants which were assessed by measuring absorbance at 695 nm. For this purpose, ascorbic acid was used as a positive control and DMSO was taken as negative control. Further, the cupric-ion assay (CUPRAC) was investigated for greenly orchestrated NiONPs [30] and the absorbance of solutions was taken at 515 nm using spectrophotometer. Moreover, the total reducing power (TRP) of greenly prepared NiONPs was studied using potassium ferricyanide method [31]. This method is based on the principle that reductones having reduction potential will reduce potassium ferricyanide (Fe3+) to form potassium ferrocyanide (Fe2+). When potassium ferrocyanide (Fe2+) reacts with FeCl_3_, it forms Fe+2-Fe+3 complex that has maximum absorption at 700 nm. The reducing power of RT-NiONPs was recorded as AAEq/mg. Further, the antioxidant scavenging potential of RT-NiONPs was evaluated. To investigate this potential, 2.4 mg DPPH was mixed with methanol (25 mL) to create a free radical environment. Further, various concentrations (1100–8.595 µg/mL) of NiONPs were prepared in DMSO and studied for their DPPH free-radical scavenging potential. The existence of reductones were determined by measuring maximum absorbance of reaction mixture at 517 nm using microplate analyzer.

(5) $$\%\;\mathrm{DPPH}\;\mathrm{scavenging}=1\;-(\frac{\mathrm{Absorbance}\;\mathrm{of}\;\mathrm{sample}}{\mathrm{Absorbance}\;\mathrm{of}\;\mathrm{control}})\;\times \;100$$

### 2.8. Enzymes Inhibition Potentials

Proteins kinase (PK) inhibition potential of *R. triquetra* synthesized NiONPs was demonstrated using actinobacterium (*Streptomyces* 85E) [32]. To confirm the PK inhibition potential, SP4 minimal media was used to acquire uniform bacterial lawns. Briefly, 100 µL inoculum was taken from standard culture and was equally spread on petri plate to achieve uniform lawns. A sterilized filter disc (6 mm) loaded with various doses of RT-NiONPs were placed on *Streptomyces* 85E painted plate. The surfactin was used as positive and DMSO as negative control. Further, petri plates were incubated (30 °C/72 h) to determine the PK inhibition potential against *Streptomyces* 85E. After incubation, different clear and bald zones were appeared. These different zones determined inhibition potentials of spores/mycelia formation in *Streptomyces* 85E strain. Finally, ZIs were measured in millimeter to determine the PK inhibition potential of RT-NiONPs.

Further, antidiabetic potency of greenly orchestrated RT-NiONPs was studied using alpha amylase (AA) inhibition assay [33]. The reaction mixture was prepared by adding starch solution (45 µL), NiONPs (15 µL), AA enzyme (30 µL), and FBS (20 µL). Further, HCL (25 µL) and iodine solutions (95 µL) were added and incubated at 50 °C for 30 min. Acarbose was used as positive and distill water as negative control respectively. The micro-plate analyzer was used to calculate the optical density at 540 nm and IC_50_ value was recorded. The % inhibition was recorded utilizing below formula:

(6) $$\%\;\mathrm{inhibition}=\frac{S\left(\mathrm{ab}\right)-\mathrm{NC}\left(\mathrm{ab}\right)}{\mathrm{Blank}\;\left(\mathrm{ab}\right)-\mathrm{NC}\left(\mathrm{ab}\right)}\times 100$$

### 2.9. Antifungal Activity

The disc-diffusion method (DDM) was used to study the antifungal potencies of RT-NiONPs using various fungal strains (*A. flavus*, *M. racemosus*, *C. albicans*, *A. niger*, *F. solani*). Fungal strains were cultured in flasks holding fungal growth media (sabouraud dextrose liquid media) (SDL) and kept in shaking incubator (37 °C/24 h). The SDL media were prepared, autoclaved, and poured in autoclaved petri plates to achieve antifungal activity. Further, 50 μL broth culture was spread on petri plate using autoclaved cotton swab to achieve uniform lawns. Filter discs (~6 mm) loaded with different concentrations (1100–34.38 μg/mL) of RT-NiONPs were kept on media plates. To compare the antifungal potential of RT-NiONPs, Amp B was taken as positive, and DMSO as negative control. Further, fungal plates were placed in an incubator for (24 h/37 °C) and zones of inhibition (ZIs) were observed with time intervals and MICs values were calculated.

### 2.10. Antibacterial Activity

To further evaluate the antimicrobial potential of RT-NiONPs, in vitro antibacterial potency was investigated using different bacterial strains, namely *P. aeruginosa*, *B. subtilis*, *S. aureus*, *K. pneumoniae*, and *E. coli*. Before antibacterial activity was investigated, bacterial cultures were refreshed in nutrient media and kept in shaking incubator at 37 °C (200 rpm /24 h). Further, bacterial strains were spread on media plates using sterilized cotton swabs. The DDM method was used to confirm the bactericidal potentials. For this purpose, 6 mm (filter disc) loaded with different concentration of RT-NiONPs (1100–34.38 μg/mL) were kept on bacterial lawns. Further, 10 µL of oxytetracycline loaded filter discs were used as positive control for five different bacterial strains. After loading test samples and positive control, petri plates were kept in incubator at 37 °C/24 h and observed after time intervals for ZIs. Finally, MICs were calculated to study the bactericidal potentials of RT-NiONPs.

## 3. Results and Discussion

**Figure 2:**
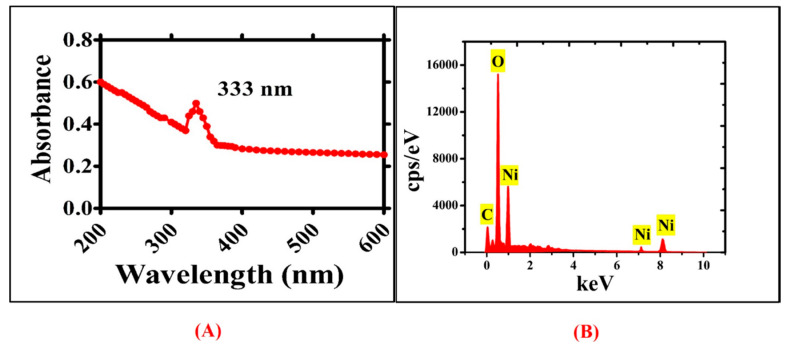
UV visible spectra and EDS analysis for RT. NiONPs (**A**) UV visible spectra for NiONPs (**B**) Elemental composition of NiONPs using EDS analysis.

The earlier research studies have confirmed the presence of different functional biomolecules such as emodin, Kaempferols-7-O-CH_3_ ether, Kaempferols-4-O-CH_3_ ether), gluside, quercetins, and physcion [24,25]. These biomolecules can act as a base source, bioreductant, stabilizers, and capping agents for the convenient synthesis of NiONPs. Previously, NiONPs have been fabricated using a variety of natural plants with potential medicinal values [26]. In the current study, NiONPs has been orchestrated using *R. triquetra* leaf extract via green method. The visual colour change from brown to greenish black revealed the formation of NiONPs. The photo-spectrometric analyses of NiONPs showed broad spectra at 333 nm (*λ*max), indicating the formation of NiONPs and validating optical observation. The UV visible spectra of green NiONPs are illustrated in Figure 2A. Elemental mapping and atomic content were confirmed using EDS analyses. Figure 2B shows EDS peak indicating strong signals at 0.94, 7.08, an 8.12 KeV for the presence of both nickel and oxygen. The presence of carbon in the spectra is ascribed to grid support. Moreover, no other peak for any elements apart from nickel ‘Ni’ and oxygen ‘O’ have been found, indicating the phase purity of greenly synthesized NiONPs.

**Figure 3:**
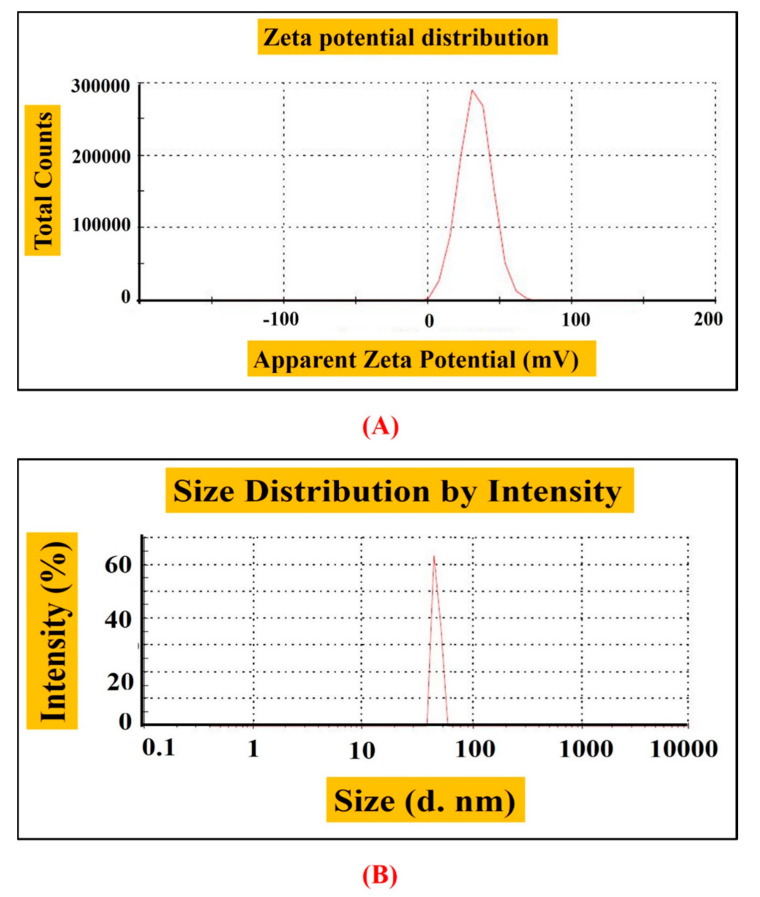
(**A**) Zeta potential of NiONPs (**B**) Size distribution of RT-NiONPs.

The hydrodynamic size and stability of RT-NiONPs were demonstrated by DLS and Zeta potential analysis. Zeta-potential or electro-kinetic potential refers to the measure of an effective surface functionality and surface charge on nanoparticle. The magnitude of zeta potential confers to particle stability. Nanoparticles with a high zeta potential exhibit increased stability, i.e., the dispersion or solution will resist the aggregation and agglomeration of nanoparticles. In our research study, data revealed a particle size of 65 nm, zeta-potential of −11 mV, and PDI of 1.000 (Figure 3A,B). Our DLS results are in agreement with previous studies using *Rhamnus virgata* mediated NiONPs [26]. DLS is mostly investigated to confirm the size of nanoscale particles in different suspensions. The mean hydro-dynamic particles diameter (d. nm) in aqueous medium show particles aggregation [11,20,34].

**Figure 4:**
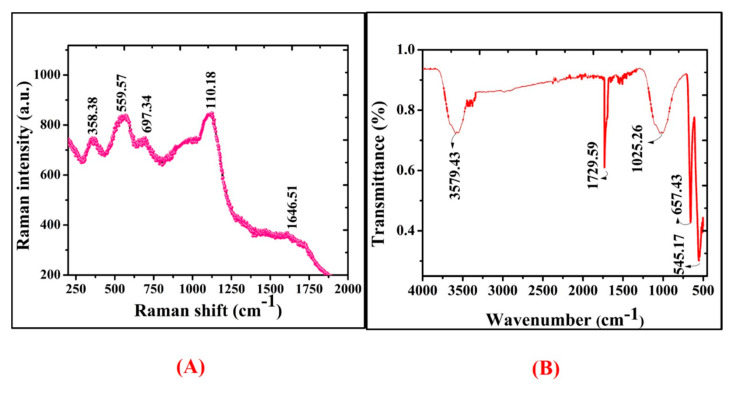
(**A**) Raman spectra of NiONPs (**B**) FT-IR spectra of biogenic NiONPs.

Raman spectral study was further used to analyze the vibrational modes of *R*. *triquetra* mediated NiONPs. Raman spectra in Figure 4A revealed the positioning of major modes at 358.38 (1 Phonon), 559.57 (1 Phonon), 697.34 (2 Phonon), 1106.18 (2 Phonon), and 1646.51 cm^−1^ (2 Magnon). The intense peaks concluded that the greenly orchestrated nanoparticles are defect rich. The broad peak attributes to the antiferromagnetic behaviour and is related to the spin of individual Ni++. This antiparallel spin behaviour of ‘Ni++’ also signifies that NiONPs are nano-size in nature (crystal size: ~25 nm). Raman shifts revealed purity and correspond to earlier research studies using *S. thea* and *G. wallichianum* orchestrated NiONPs [7,35]. The difference in Raman scattering peaks might be due to the relative positions, size, intensity and effects of stress and strain [36]. Further, FT-IR analysis was performed to determine the qualitative distribution of functional groups adsorbed on the surface of NiONPs. The infrared absorption bands in Figure 4B revealed significant vibrations at 545.17 cm^−1^ and 657.43 cm^−1^ (Ni-O vibrations in stretching mode), 1025.26 cm^−1^ (Ethers = C-O-C symmetric stretching), 1729.59 cm^−1^ (Aldehyde group of carbonyl ‘-CHO’), and 3579.43 cm^−1^ (alcohols and phenols of OH stretching). According to previously reported research work, IR bands between 470 and 800 cm^−1^ indicate Ni-O vibrations in the stretching mode [37,38].

**Figure 5:**
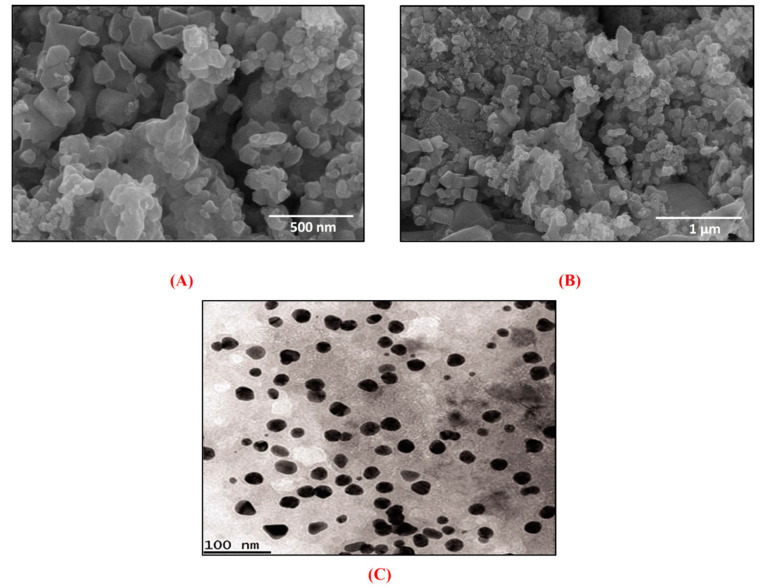
(**A**,**B**) Various SEM images of RT-NiONPs using nickel nitrate salt as a precursor (**C**) TEM images of RT- NiONPs.

In addition, SEM analysis was performed to know the shape and surface morphology of greenly fabricated NiONPs. Figure 5A,B depicts SEM images of NiONPs confirming the spherical/agglomerated shape of NiONPs. The crystallographic structure and accurate particle size of the biogenic NiONPs were studied by TEM analysis (Figure 5C).

**Figure 6:**
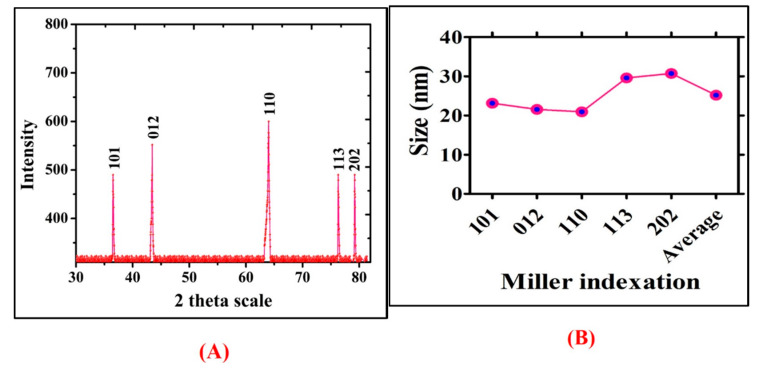
(**A**) XRD Spectra of the RT-NiONPs (**B**) Size calculation and Miller indexation.

Moreover, phase structure of NiONPs was assessed by XRD analyses. The XRD pattern of the synthesized NiONPs and miller indexation have been illustrated in Figure 6A,B, which indicates the diffraction bands at 36.52 (101), 43.44 (012), 63.11 (110), 76.28 (113), and 79.2 (202), corresponding to fcc symmetry in NiONPs crystalline lattice. Previously, Khalil et al. [35] and Iqbal et al. [26] have synthesized biogenic NiONPs from *Sageretia thea* and *Rhamnus virgata* leaf extracts and reported similar results.

### 3.1. Antimicrobial Potentials

**Figure 7:**
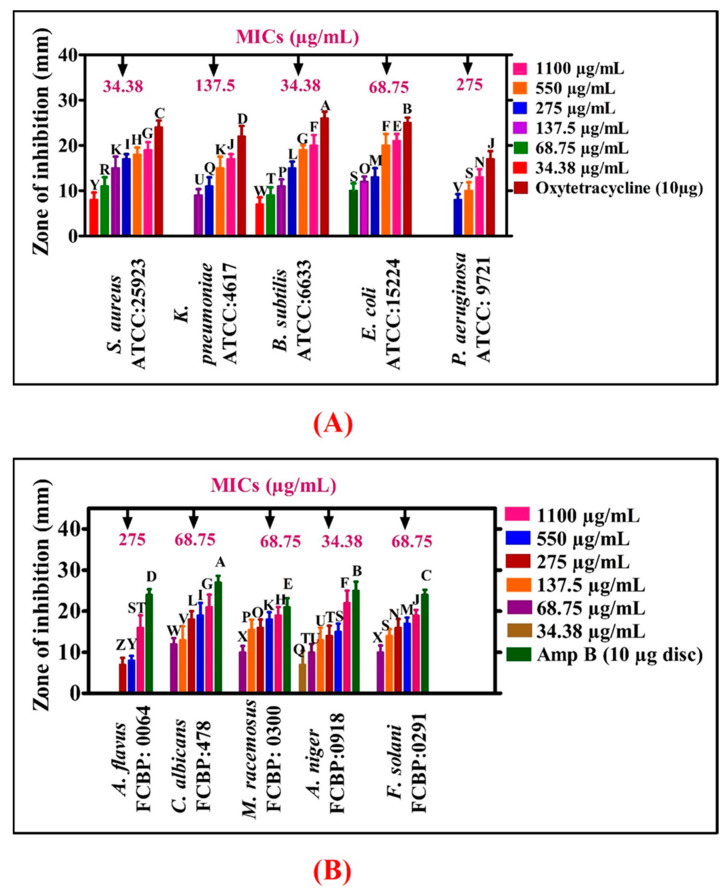
(**A**) Various antimicrobial activities of RT mediated NiONPs. Data represents the mean of three replicates and each alphabet indicates significance at *p* < 0.05 (**A**) MICs values of RT-NiONPs against various pathogenic bacterial strains (**B**) Antifungal potencies of RT-NiONPs against different fungal strains.

The antibacterial activities of NiONPs were demonstrated against different bacterial strains (*E. coli*, *P. aeruginosa*, *B. subtilis*, *K. pneumoniae*, *S. aureus*) in concentrations ranging from 34.38 to 1100 µg/mL. Most of the BSs were found susceptible using NiONPs and have shown significant antibacterial activities. Different MIC values were calculated for different bacterial strains *P*. *aeruginosa* (275 µg/mL), *K. pneumoniae* (137.5 µg/mL), *E. coli* (68.75 µg/mL), and *S. aureus* and *B. subtilis* (34.38 µg/mL). *S. aureus* and *B. subtilis* were found to be the most susceptible strains with MIC: 34.38 µg/mL while *P*. *aeruginosa* was found to be the least susceptible strain (MIC: 275 µg/mL), as shown in Figure 7A. Oxytetracyclines was taken as positive control and no single concentration of NiONPs determined a stronger potential than Oxytetracyclines. Overall, NiONPs have determined significant antibacterial activities which are in agreement with previous studies of greenly orchestrated NiONPs using *G*. *wallichianum* and *R*. *virgata* [7,26]. The strong bactericidal potency of NiONPs might be due to biomolecules adsorbed on NPs surface. In conclusion, RT-NiONPs have shown dose-dependent results. Some studies have explained that the bactericidal potential of NPs is due to ROS generation. Further, NPs damage membrane (membrane proteins) and result in bacterial cell death. Similarly, surface defects in the symmetry of NPs is responsible for the inhibition of bacteria and result in cell damage [39].

Numerous research studies have been conducted on the bactericidal potential of NiONPs while only limited research work has been published on fungicidal activities of NPs. In the present study, the fungicidal potency of RT-NiONPs was investigated using different fungal strains (FS). The different FS such as *F. solani*, *M. racemosus*, *A. niger*, *A. flavus* and *C. albicans* were exposed to different concentration of RT-NiONPs (34.38–1100 µg/mL) (Figure 7B). Amp-B was taken as positive control to confirm the inhibition potential of RT-NiONPs. According to our literature review, the current study, for the first time, reported the antifungal potential of RT-NiONPs. Our *R*. *triquetra*-NiONPs revealed a concentration-dependent inhibition response against different fungal strains where *A. flavus* was the least susceptible fungal strain (MIC: 275 µg/mL while *A*. *niger* was the most susceptible strain (MIC: 34.38 µg/mL). Previously, concentration mediated fungicidal activities were reported using different fungal strains [7] and are in line with our presently synthesized RT-NiONPs. The MIC values for different pathogenic bacterial and fungal strains are provided in Figure 7.

### 3.2. Antileishmanial Potentials

**Figure 8:**
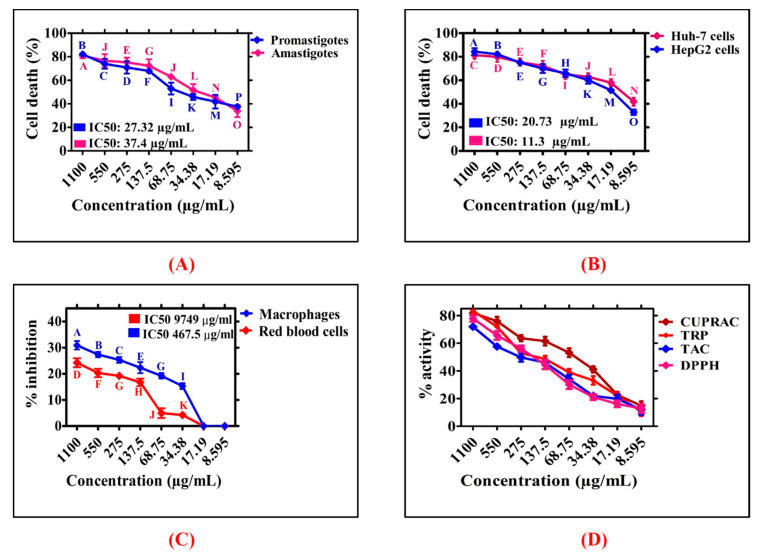
Biocompatibility, cytotoxic and antioxidant properties of NiONPs. Data represents the mean of three replicates and each alphabet indicates significance at *p* < 0.05 (**A**) Antileishmanial activities against Leishmanial parasites (**B**) Anticancer potentials of RT-NiONPs against HUH-7 and HepG2 cell lines (**C**) Biocompatibility of NiONPs against RBCs and Macrophages (**D**) Antioxidant activities of RT-NiONPs.

Leishmaniasis is a widespread tropical disease caused by leishmanial parasites [27]. The drug antimonial was developed as a potential candidate to cure leishmaniasis but has lost its therapeutic potential as they have developed resistance. Thus, scientists are involved in designing alternative routes to fight and manage this global disease. Therefore, extensive research studies are needed to design some novel and effective nanomaterials. Various nanomaterials are being used to study their antileishmanial potential [11,14]. However, greenly orchestrated NiONPs are rarely studied to investigate their cytotoxic potential. In current study, antileishmanial potentials of *R. triquetra* orchestrated NiONPs was investigated against *L*. *tropica*. The parasites were treated with different doses of RT-NiONPs (8.595−1100 µg/mL) (Figure 8A). The antileishmanial potential increased with RT-NiONPs, thus indicating a dose-dependent response. The RT-NiONPs displayed significant potential against *L*. *tropica* promastigote (IC_50_: 27.32 μg/mL). Similarly, the antileishmanial potential of RT-NiONPs was reported against *L*. *tropica* amastigotes (IC_50_: 37.4 μg/mL). Our results of RT-NiONPs are in agreement with the previous reports of *Sageretia thea* mediated NiONPs [35].

### 3.3. Anticancer Potential of NiONPs

Among the different types of cancer, liver cancer is the second deadliest cancer in males and sixth deadliest in females [15,40,41]. Different risk factors are involved in regulating the rate of cancer such as viral infections, heavy consumption of alcohol and toxin exposures (aflatoxin). In the current study, the anticancer potential of greenly orchestrated RT-NiONPs was investigated against HUH-7 and HepG2 liver cancer cell lines using an MTT assay [28]. To achieve this purpose, HUH-7 and HepG2 cancer cells were exposed to different concentrations of RT-NiONPs (1100–8.595 μg/mL) as summarized in Figure 8B. The NiONPs have determined strong reduction in metabolic activities of both HUH-7 and HepG2 cancer cells using different doses of NiONPs. The metabolic activities were decreasing while increasing RT-NiONPs concentration. The highest anticancer activity recorded was 81.41% for HuH-7 and 84.41% for HepG2 at 1100 µg/mL and anticancer activity decreased with NiONPs concentration. Further, IC_50_ values were recorded for RT-NiONPs which are 11.3 µg/mL for (HuH-7) and 20.73 µg/mL (HepG2) cell lines respectively. The anticancer potential induced by RT-NiONPs even at low concentration (8.595 µg/mL) could be due to different functional molecules adsorbed from leaves broth on the surface of NiONPs. The reduction in the metabolic activities have determined that RT-NiONPs have strong anticancer activities. The results of RT-NiONPs using HUH-7 and HepG2 are in correspondence with the previously published reports using *G*. *wallichianum* and *Euphorbia heterophylla* [7,42].

### 3.4. Biocompatibility Assays with Human Red Cells and Macrophages

Considering the interest in biomedical applications, the biosafety and biocompatible nature of RT-NiONPs were investigated using previously optimized protocol [7]. According to biosafety principle guidelines, biological and chemical substances having hemolysis >5% are hemolytic, 2−5% are slightly hemolytic, while <2% is non-hemolytic [43]. If the tested NPs are hemolytic, it will damage erythrocytes and result in hemoglobin release from RBCs. To confirm the hemolytic potential, red cells were treated with different doses of RT-NiONPs (1100−8.595 µg/mL) and revealed dose dependent response. The hemoglobin release was 24.23% at highest concentrations of 1100 µg/mL (Figure 8C). Research studies concluded that RT-NiONPs are non-hemolytic at 17.19 µg/mL, slightly hemolytic at 68.75 µg/mL, and hemolytic at >68.75 µg/mL. On the whole, RT-NiONPs are non-toxic and biocompatible at low concentration against red cells. Our biocompatibility results of RT-NiONPs are in line with the previously synthesized *S. thea* and *G*. *wallichianum* mediated NiONPs [7,35].

The biosafe and biocompatible nature of RT-NiONPs was further determine using normal human macrophages (HM). For this purpose, confluent HM cells were seeded in sterilized 96-well plate containing RPMI media and were cultured for 24 h. Further, the seeded cells were exposed to different doses of RT-NiONPs (1100−8.595 µg/mL). Further, an MTT cell viability assay was performed to confirm the biosafe nature of RT-NiONPs. The results shown in Figure 8C indicate that biosynthesized RT-NiONPs at 1100 µg/mL inhibited the growth of HM cells by ~30.89% confirming its biosafe nature, thus indicating a dose-dependent response. Normally, HM cells have developed a natural strategy to neutralize ROS produced from external sources. Previous research studies reported that ROS are non-toxic to both red cells and HM cells at low concentrations unless concentrations increase beyond the limit, which will result in toxicity to both erythrocytes and macrophages [44]. Previously, Iqbal et al. [26] reported the biocompatibility potential of greenly fabricated NiONPs against HM cells using *Rhamnus virgata*.

### 3.5. Antioxidant Activities

The antioxidant potential of phytogenic NiONPs was evaluated (Figure 8D). The maximum value for TAC of *R. triquetra* mediated NiONPs in terms of AA Emg^−1^ was reported as 71.93% at 1100 µg/mL. Generally, a TAC assay is performed to evaluate the scavenging potential of reductones/antioxidants present in the test sample towards ROS species. Our TAC results are in correspondence to Abbasi et al. [7] using *Geranium wallichianum* mediated NiONPs. Further, a cupric-ion assay was investigated to assess the scavenging potentials of antioxidants species adsorbed on the surface of RT-NiONPs. The maximum score for cupric ion assay of green RT-NiONPs was obtained as 81.83%. According to our literature review, a cupric-ion assay was demonstrated for the first time on RT-NiONPs. Moreover, greenly orchestrated NiONPs were further explored to determine the surface adsorbed antioxidant molecules. To achieve this goal, TRP assay was performed. The maximum value for TRP was recorded as 83.41% at 1100 µg/mL which is corroborated with the earlier research report using *Rhamnus virgata* mediated NiONPs [26]. Further, DPPH free radical scavenging assay was demonstrated to assess the presence of radical scavengers (antioxidants) adsorbed on the surface of greenly fabricated NiONPs. The highest DPPH value reported is 77.91% and our DPPH data are in agreement with the previous studies using *Sageretia thea* mediated NiONPs [35]. Together, the antioxidant potential confirmed the presence of radical scavengers on RT-NiONPs which play a potential role in the stabilization, reduction, and capping of nano nickel oxide particles [7].

### 3.6. Enzymes Inhibition Potentials

**Figure 9:**
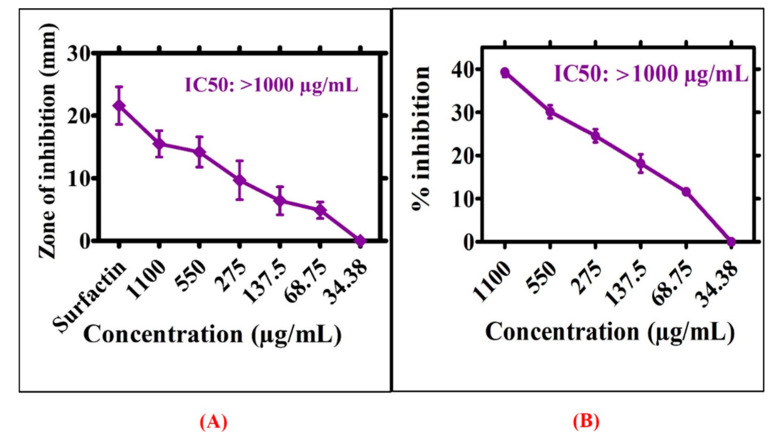
(**A**) Inhibition potential against protein kinase of RT orchestrated NiONPs (**B**) Inhibition potential against alpha amylase.

Greenly orchestrated RT-NiONPs were examined for their protein kinase (PKs) inhibition activity. Figure 9A shows the significant PK inhibition potential of *R. triquetra* mediated NiONPs using different doses of NiONPs (34.38–1100 μg/mL). Moderate PK inhibition potential was revealed for RT-NiONPs. The maximum value for ZIs was 15.5 mm with IC_50_ > 1000 μg/mL. On the whole, ZIs obtained for greenly orchestrated NiONPs were smaller as obtained from surfactin (positive control). These results suggested cell viability at lower concentrations of NiONPs, consistent with a previous research report using *G. wallichianum−*NiONPs [7]. Further, A-amylase assay was demonstrated to evaluate the inhibition potentials of *R. triquetra*-NiONPs using different concentrations (1100−34.38 µg/mL). The biogenic NiONPs were observed to cause increased % inhibition (39.31%) at 1100 µg/mL (Figure 9B). However, the percent inhibition significantly decreased with a decrease in concentration. Overall, a moderate inhibition potential is reported. The results of our current report are in agreement with a previous research study using *S*. *thea* mediated NiONPs [35].

## 4. Conclusions and Future Directions

This study has established a simple, eco-friendly, and economically viable method to synthesize NiONPs simply by mixing NiNO3 with aqueous broth of *R*. *triquetra* leaves which are free of toxicants and rich in functional biomolecules. The actions of different functional biomolecules in the leaves extract may result in the reduction, stabilization, and capping of NiONPs. The microscopic analyses from SEM and TEM confirmed the predominant spherical shape and small size of NiONPs (~25 nm). Further, spectroscopic studies from UV–vis, Raman, FT-IR, EDX, zeta potential, and DLS supported the fabrication and stability of NiONPs. Significant anticancer potentials were revealed against different cancer cell lines (HepG2: IC_50_: 20.73 and HuH-7: IC_50_: 11.3 µg/mL). Further, antileishmanial potential was investigated against leishmanial parasites (promastigotes; IC_50_: 27.32, amastigotes: IC_50_: 37.4 µg/mL). The outcomes of a biocompatibility assay revealed that NiONPs are non-toxic and biocompatible. NiONPs determined significant free radical scavenging and moderate enzyme inhibition activities. Further, NiONPs determined significant antimicrobial studies against different bacterial and fungal strains. In conclusion, our results unequivocally indicate that RT-NiONPs may be used as a safer alternative in biotechnological, biomedical, and pharmaceutical industries. Further, more in vitro and in vivo studies are recommended in different animal models before bringing NiONPs into clinical trials.
